# Trends in myopia development among Chinese children and adolescents in Xuzhou during one academic year

**DOI:** 10.3389/fmed.2024.1391269

**Published:** 2024-07-10

**Authors:** Lin Li, Ya Liao, Qian Wang, Mei Wang, Wenxuan Zhang, Xiaojuan Wang

**Affiliations:** ^1^School of Medical Technology, Xuzhou Medical University, Xuzhou, China; ^2^Department of Ophthalmology, The First People’s Hospital of Xuzhou, The Affiliated Xuzhou Municipal Hospital of Xuzhou Medical University, Xuzhou, China

**Keywords:** myopia, development, children, adolescents, prevalence

## Abstract

**Purpose:**

This study investigates the prevalence and progression of myopia among primary and secondary school students in Xuzhou City, China, during one academic year.

**Methods:**

The study employed a prospective research design and utilized a whole-group sampling method to conduct non-cycloplegic spot photo screenings on 37,938 students from 44 primary and secondary schools in Xuzhou City, China. A one-year study was conducted to gather spherical equivalent refraction (SER), and subsequent analysis was carried out to explore the disparities in myopia prevalence among primary and secondary school students within the same academic year, as well as the progression of myopia.

**Results:**

During the 2022 academic year, the overall prevalence of myopia in the first and second semesters was 62.6 and 64.2% respectively, indicating an increasing trend. Particularly in primary school (Grades 1–6), the prevalence of myopia increased with higher grade levels, and significant variations in myopia prevalence were observed mainly in grades 1–3 and 7 (*p* < 0.05). The incidence rate of myopia in middle school remained stable, while in primary school, there was a positive correlation between myopia incidence and the grade level, with the highest rate of 20.1% in grade 6. Among the myopic population, the median value of spherical equivalent refraction slightly decreased between the two semesters. The proportion of high myopia increased among students in grades 5–8.

**Conclusion:**

Our study revealed that within one academic year, the prevalence of myopia and the severity of myopia have significantly increased in Xuzhou City, China, accompanied by an increase in the proportion of high myopia. For different grade levels, we should adopt personalized prevention and control measures, with a particular focus on lower grade levels and students who have just entered a new grade.

## Introduction

Myopia is a common condition that causes visual impairment and is a major public health problem, especially among children and adolescents (1). In the absence of effective preventive and control measures, it is projected that the global myopia population will approach approximately 5 billion by the year 2050, with high myopia affecting approximately 10% of the global population. East Asia and Southeast Asia are identified as regions with the highest prevalence of myopia, where the occurrence among young adults ranges from 80 to 90%. Furthermore, the prevalence of high myopia among this demographic in these regions is relatively elevated, ranging from 10 to 20%. High myopia substantially increases the risk of ocular complications, encompassing myopic maculopathy, retinal detachment, glaucoma, cataracts, and a range of other pathologies. These conditions have the potential to cause enduring and irreversible visual impairment (2, 3). The global cost of myopia interventions is also rising (4).

Based on research surveys (5–10), there has been a consistent rise in the prevalence of myopia among children and adolescents in China, indicating a severe situation regarding myopic incidence. However, current longitudinal studies on this topic, both in China and other countries (5–8, 10), typically have a minimal time interval of 1 year. Nonetheless, several findings have found a significant prevalence in the incidence and progression of myopia within a single academic year (two semesters) among students, particularly in higher grades burdened with heavier academic loads. Consequently, we undertook a 1-year study, meticulously examining the myopia status of primary and secondary school students in Xuzhou City throughout two semesters. The primary objective was to assess the incidence and developmental trends of myopia among these students within the same academic year. Gaining insights into the patterns of myopia incidence and development among children and adolescents is imperative for refining and adapting local measures aimed at preventing and controlling myopia.

## Methods

### Study population

In accordance with the requirements of the “Comprehensive Control of Myopia in Children and Adolescents” program (11), Xuzhou City has implemented comprehensive vision screening for primary and secondary school students since the beginning of 2019. The screening took place twice a year, within 1 month after the commencement of each semester (6 months). This study employed a whole-group sampling method to select 44 schools from five districts in Xuzhou City randomly. The survey data collected from the same cohort of students during both the first and second semesters of the 2022 academic year were analyzed. A total of 38,041 students participated in the research, with individuals wearing contact lenses or affected by other conditions that could potentially impact ocular data collection being excluded from the study. Ultimately, 37,938 students were included in the final analysis. This study followed the principles of the Helsinki Declaration and was approved by the Ethics Committee of Xuzhou Medical University Xuzhou Municipal Hospital, China (No. Xyll[2019]022).

### Data collection

A week before the initiation of the screening process, thorough communication was conducted with the schools to acquire comprehensive demographic information about the students, encompassing their names, genders, ages, and other pertinent particulars. It was explicitly communicated that participants were strictly prohibited from wearing contact lenses on the examination day. Furthermore, a comprehensive briefing was provided to all participants and their respective guardians, elucidating the study’s purpose, extent, and significance. Before the examination, explicit verbal consent was obtained from the guardians to ensure their understanding and agreement with the research protocol.

Subjects were screened for refractive error using the Spot photo screener (Welch Allyn, VS100) for non-cycloplegic photorefraction. The screening was performed by experienced ophthalmic staff, including one ophthalmologist and five optometrists. Before the refractive screening, subjects underwent a slit lamp examination and were asked about their ocular history to exclude those with cataracts or other conditions that may affect refraction, as well as those who had undergone refractive surgery or wore contact lenses. Subjects with a history of ortho-k lenses within the last month were also recorded. The examiner used the Spot photo screener to collect data from the subjects in a relative darkroom at a distance of approximately 1 m in the uncorrected state. The data was automatically generated and uploaded to the terminal for recording. If significant refractive errors, strabismus, or anisometropia were detected, feedback was provided to the school and parents with a recommendation for further examination at a medical clinic.

### Definition

The spherical equivalent refraction (SER) is calculated by adding the sum of the sphere power with half of the cylinder power. If the result exceeds the ±7.50 D range of the Spot screener, it is recorded as ±8.00D. If the SER of either eye is less than or equal to −0.50 D, the student is classified as myopic. Individuals who wear ortho-k lenses are also considered myopic (12). The degree of myopia is classified into mild myopia (−3.00 D < SER ≤ −0.50 D), moderate myopia (−6.00 D < SER ≤ −3.00 D), and high myopia (SER ≤ −6.00 D).

### Statistical analyses

The statistical analysis was conducted using SPSS 27.0 (IBMSPSS, Armonk, NY, United States), Microsoft Excel 2010, and the figures were prepared using OriginPro 2021. The statistical tests were two-sided, and a *p*-value < 0.05 was considered statistically significant. The skewed distribution of continuous variables was represented by the median (interquartile range, IQR), while count data was presented as frequency (rate). The Mann–Whitney U test was used to compare differences between the two groups, and the chi-square test was used to compare the prevalence and incidence of myopia among different grade levels. Spearman rank correlation test was used to assess the correlation between the spherical equivalent refraction (SER) of the left and right eyes. Due to the high correlation between the SER of both eyes (Spearman’s rank correlation = 0.932, *p* < 0.01), we used the SER of the right eye to evaluate the progression of myopia in students.

## Results

### General characteristics

**Figure 1:**
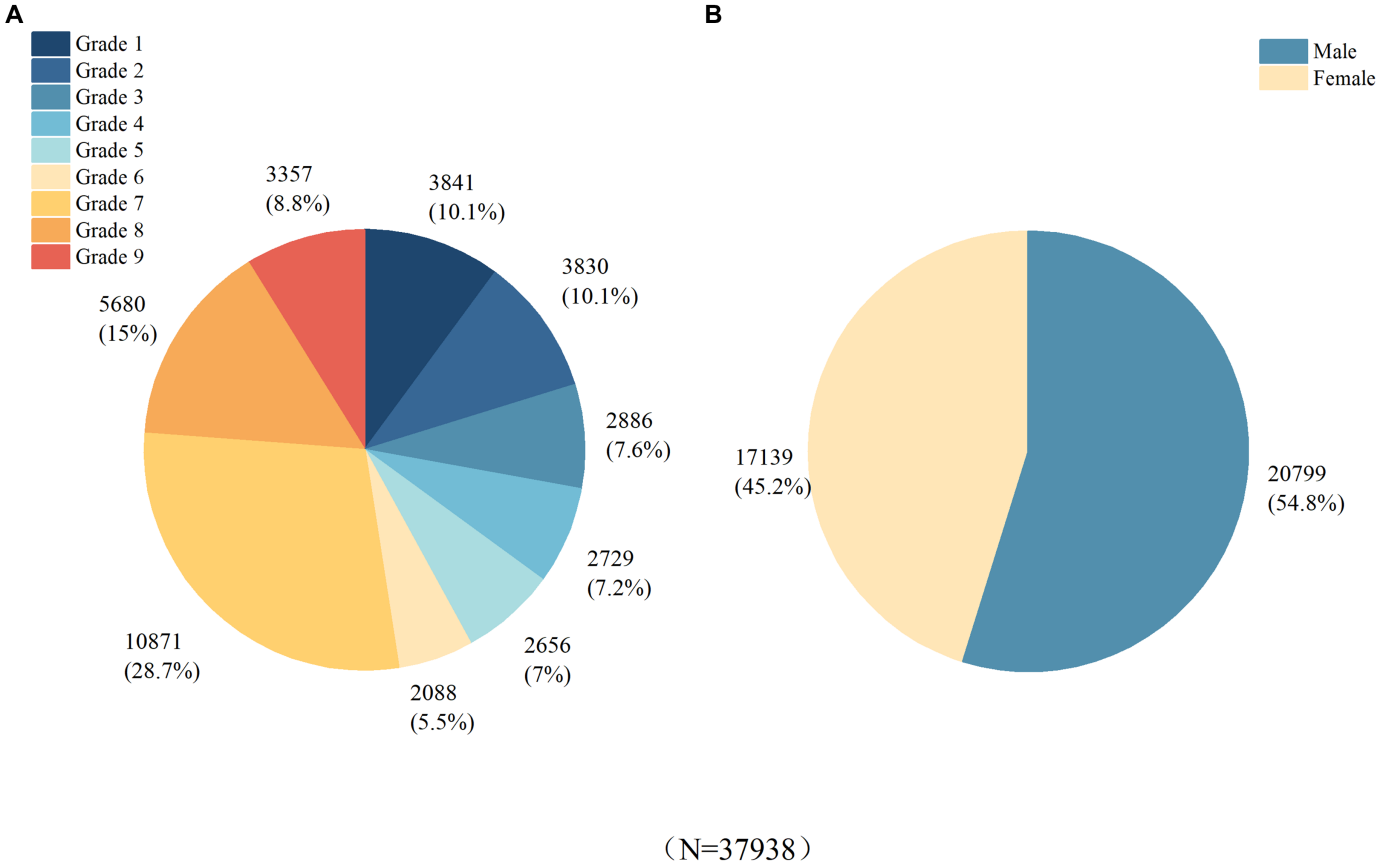
Basic Characteristics of the Study Population. **(A)** Distribution of subjects by grade level. **(B)** Distribution of subjects by gender.

The study encompassed a cohort of 38,041 students enrolled in 44 schools. A total of 104 participants were excluded from the analysis due to specific criteria, including the use of contact lenses, recent ortho-k lens wear, prior eye surgeries, or the presence of other ocular conditions that could potentially influence the refractive status. Consequently, the final dataset comprised 37,938 eligible records meeting the inclusion criteria. Among these records, 20,799 (54.8%) corresponded to male students, while 17,139 (45.2%) represented female students. The distribution of the sample size, grade levels, and gender can be found in Figure 1.

### Myopia prevalence and incidence

Table 1 presents the changes in the prevalence of myopia among students in grades 1–9 during the first and second semesters of the 2022 academic year, including both male and female students. The overall myopia prevalence for the 2022 academic year was 62.6 and 64.2%, showing an increasing trend. Within the same semester, there was a linear relationship between myopia prevalence and grade, with an increase in myopia prevalence as the grade level advanced during the primary school stage. The variation in myopic prevalence among different grades is mainly concentrated in grades 1–3 and 7 (*p* < 0.05). Among them, the largest increase in myopia prevalence is observed in grade 3 (2.8%), followed by grade 2 (2.6%), grade 1 (2.2%), and grade 7 (1.4%). The myopia prevalence in both males and females increases from the beginning and end of the same academic year, with females having a higher myopia prevalence than males.

**Table 1:** Myopia prevalence by grade and gender (%).

	First semester	Second semester	*p*
**Grades**			
1	37.7%	39.9%	0.044*
2	50.4%	53.0%	0.028*
3	49.2%	52.0%	0.033*
4	58.6%	60.9%	0.087
5	68.0%	68.7%	0.555
6	76.7%	77.7%	0.417
7	68.6%	70.0%	0.024*
8	73.6%	74.4%	0.325
9	68.3%	70.1%	0.119
*p*	<0.001*	<0.001*	
**Gender**			
Male	59.7%	61.3%	<0.001*
Female	66.1%	67.7%	0.002*
*p*	<0.001*	<0.001*	
Total	62.6%	64.2%	<0.001*

This table presents the prevalence of myopia, stratified by both grade level and gender.

*Significance was set at *p* < 0.05.

The incidence rate of myopia during the academic year of 2022 exhibited notable variations primarily among primary school students, while the incidence rate remained relatively stable among middle school students. Within the primary school stage, there was a positive correlation between the incidence of myopia and grade level, with the highest incidence rate of 20.1% observed in the 6th grade. Moreover, there was no statistically significant difference in myopia incidence between males and females (Table 2).

**Table 2:** The incidence rate of myopia in the 2022 academic year (%).

	Myopia incidence	*p*
**Grades**		
1	8.4%	**<0.001** ^a*^
2	10.6%	
3	13.3%	
4	14.8%	
5	13.5%	
6	20.1%	
1–6	11.9%	
7	15.1%	0.617^b^
8	16.1%	
9	15.7%	
7–9	15.4%	
Gender		0.345
Male	15.1%	
Female	16.0%	
Total		<0.001*

*Significance was set at *p* < 0.05.

a, b represent *p*-values for comparisons between myopia incidence in primary and junior high school grades, respectively.

### Refractive error

**Figure 2:**
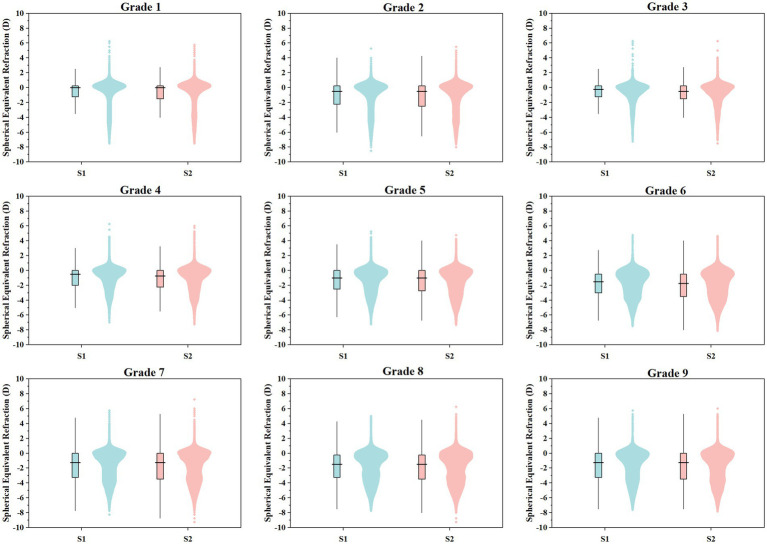
Distribution of spherical equivalent refraction in subjects from Grade 1 to Grade 9 during the 2022 academic year. S1 denotes First Semester, S2 denotes Second Semester.

Table 3 shows the spherical equivalent refraction (SER) of primary and secondary school students throughout the 2022 academic year, encompassing the first and second semesters. Within the myopic population (identified as myopic during the initial semester screening), the median SER values exhibited a slight decline during both halves of the year. Nonetheless, the extent of this change was relatively modest. Figure 2 illustrates the distribution of spherical equivalent refraction (SER) for children and adolescents in grades 1–9 during the 2022 academic year.

**Table 3:** SER values for different grade levels in the 2022 academic year (D).

	No myopia			Myopia		
	First semester	Second semester	*p*	First semester	Second semester	*p*
**Grades**						
1	+0.25 (0.00,+0.50)	+0.25 (0.00,+0.50)	0.001*	−2.75 (−4.25, −1.00)	−2.75 (−4.25, −1.25)	<0.001*
2	+0.25 (0.00,+0.50)	+0.25 (0.00,+0.50)	<0.001*	−2.50 (−4.25, −1.00)	−2.75 (−4.50, −1.25)	<0.001*
3	+0.25 (0.00, +0.25)	+0.25 (0.00, +0.50)	<0.001*	−1.50 (−2.75, −0.75)	−1.75 (−3.00, −1.00)	<0.001*
4	0.00 (0.00, +0.25)	0.00 (−0.25, +0.50)	<0.001*	−1.75 (−3.00, −1.00)	−2.25 (−3.25, −1.25)	<0.001*
5	0.00 (0.00, +0.25)	0.00 (−0.25, +0.25)	<0.001*	−2.00 (−3.25, −1.00)	−2.25 (−3.75, −1.25)	<0.001*
6	0.00 (−0.25, +0.25)	0.00 (−0.25, +0.25)	<0.001*	−2.25 (−3.75, −1.25)	−2.50 (−4.00, −1.25)	<0.001*
7	+0.25 (0.00, +0.50)	+0.25 (−0.25, +0.50)	<0.001*	−2.50 (−4.00, −1.25)	−3.00 (−4.25, −1.50)	<0.001*
8	+0.25 (0.00 + 0.50)	+0.00 (−0.25 + 0.50)	<0.001*	−2.75 (−4.00, −1.25)	−3.00 (−4.25, −1.50)	<0.001*
9	+0.25 (0.00 + 0.50)	+0.25 (−0.25 + 0.50)	<0.001*	−2.50 (−4.00, −1.25)	−2.75 (−4.25, −1.25)	<0.001*
Total	+0.25 (0.00 + 0.50)	+0.25 (0.00 + 0.50)	<0.001*	−2.50 (−3.75, −1.25)	−2.75 (−4.00, −1.25)	<0.001*
*p*	<0.001*	<0.001*		<0.001*	<0.001*	

*Significance was set at *p* < 0.05.

### Fluctuations in the severity of myopia

**Figure 3:**
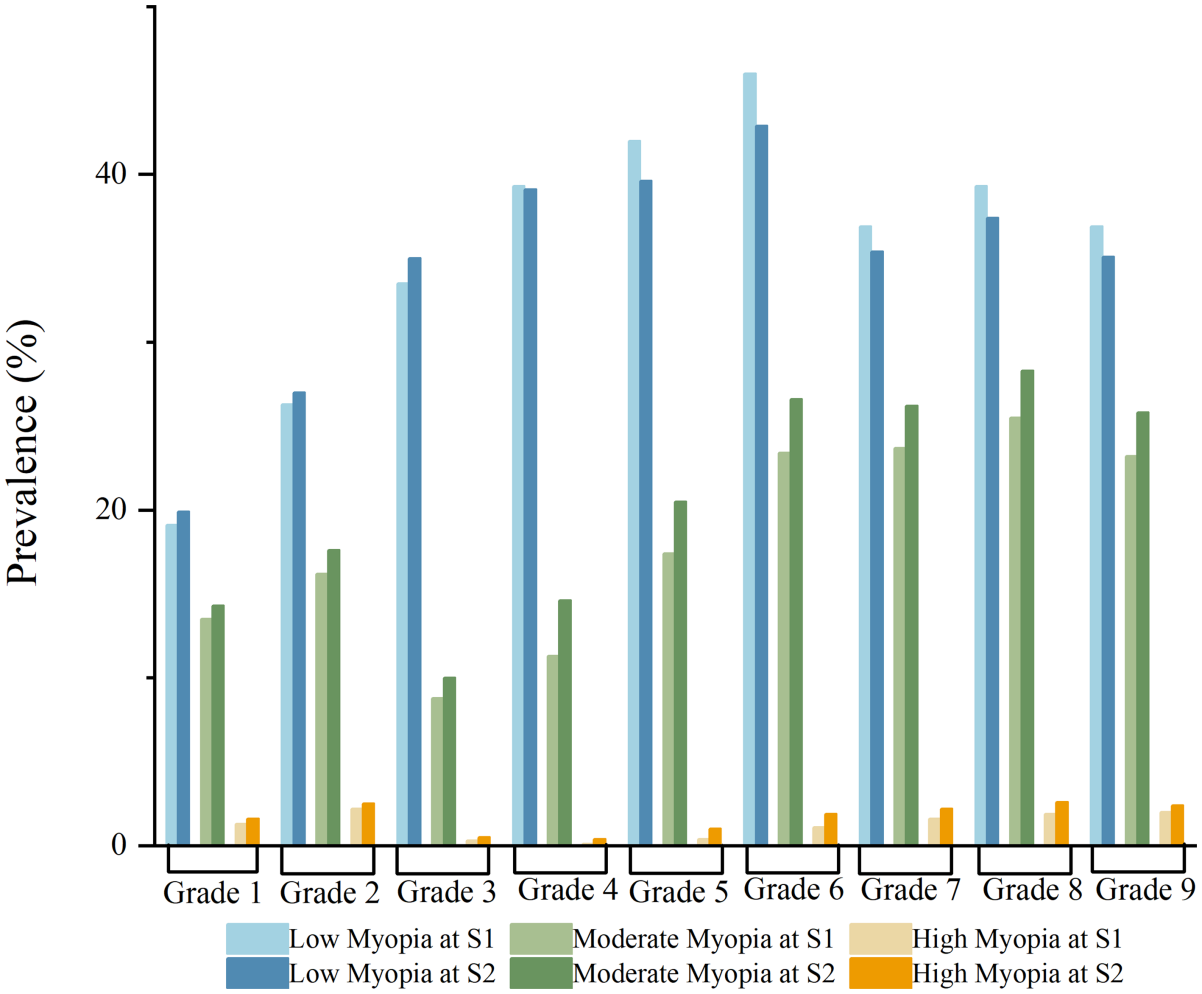
Distribution of myopia rates across different grade levels in primary and secondary schools during the 2022 academic year. Comparison of myopia rates between the first semester (S1) and the second semester (S2).

Further analysis showed that the proportion of mild myopia in grades 6–8 decreased from semester 1 to 2 (46.0, 36.9, 39.3% vs. 42.9, 35.4, 37.4%), while the proportion of moderate myopia increased in grades 4–9 (11.3, 17.4, 23.4, 23.7, 25.5, 23.2% vs. 14.6, 20.5, 26.6, 26.2, 28.3, 25.8%). In addition, there was an increase in the proportion of high myopia in grades 5–8 (0.4, 1.1, 1.6, 1.9% vs1.0, 1.9, 2.2, 2.6%) (Table 4; Figure 3).

**Table 4:** Changes in the degree of myopia in the 2022 academic year (%).

Grades	2022 S1				2022 S2				*p*
	No myopia	Mild myopia	Moderate myopia	High myopia	No myopia	Mild myopia	Moderate myopia	High myopia	
1	66.1%^a^	19.1%^a^	13.5%^a^	1.3%^a^	64.3%^a^	19.9%^a^	14.3%^a^	1.6%^a^	0.329
2	55.2%^a^	26.3%^a^	16.2%^a^	2.2%^a^	52.9%^b^	27.0%^a^	17.6%^a^	2.5%^a^	0.186
3	57.3%^a^	33.5%^a^	8.8%^a^	0.3%^a^	54.5%^b^	35.0%^a^	10.0%^a^	0.5%^a^	0.113
4	49.2%^a^	39.3%^a^	11.3%^a^	0.1%^a^	45.9%^b^	39.1%^a^	14.6%^b^	0.4%^a^	<0.001*
5	40.2%^a^	42.0%^a^	17.4%^a^	0.4%^a^	38.9%^a^	39.6%^a^	20.5%^b^	1.0%^b^	0.002*
6	29.5%^a^	46.0%^a^	23.4%^a^	1.1%^a^	28.6%^a^	42.9%^b^	26.6%^b^	1.9%^b^	0.007*
7	37.8%^a^	36.9%^a^	23.7%^a^	1.6%^a^	36.2%^b^	35.4%^b^	26.2%^b^	2.2%^b^	<0.001*
8	33.2%^a^	39.3%^a^	25.5%^a^	1.9%^a^	31.7%^a^	37.4%^b^	28.3%^b^	2.6%^b^	<0.001*
9	37.9%^a^	36.9%^a^	23.2%^a^	2.0%^a^	36.6%^a^	35.1%^a^	25.8%^b^	2.4%^a^	0.040*
Total	43.8%^a^	35.2%^a^	19.7%^a^	1.4%^a^	42.0%^b^	34.2%^b^	22.0%^b^	1.9%^b^	<0.001*

*Significance was set at *p* < 0.05.

First Semester (S1); Second Semester (S2).

In the population with the same level of myopia within the same grade, different superscript letters indicate.

Significant differences between semesters (*p* < 0.05).

## Discussion

Our research has unveiled that within the educational landscape in China, the prevalence and incidence of myopia among primary and secondary school students are far from desirable, and the situation is exacerbated with higher grade levels. Employing a longitudinal approach, we monitored the myopia trajectory of students spanning grades 1–9 in Xuzhou, China, throughout a single academic year encompassing both the initial and subsequent semesters. Unlike the majority of studies that typically have a one-year interval between examinations, our research findings revealed a significant progression and increase in the incidence, prevalence, and severity of myopia among children and adolescents within a single semester (6 months).

Our research investigation revealed that the prevalence of myopia among primary and secondary school students in Xuzhou City during the first and second semesters of the 2022 academic year was 62.6 and 64.2%, respectively. Notably, there was a significant increase in myopia prevalence during the second semester compared to the first semester. Furthermore, consistent with findings from both domestic and international surveys, the myopia rate among females was higher than that among males (13–15). This trend may be attributed to the relatively lower amount of time females spend engaging in outdoor activities compared to males, and longer periods of close-eye use (16, 17). Research on the prevalence of myopia, both nationally and internationally, was mostly based on cross-sectional studies with a minimum time interval of 1 year. There is a paucity of longitudinal studies focusing on short-term changes in myopia. According to a meta-analysis of myopia prevalence among children and adolescents in China from 1998 to 2016, the overall myopia prevalence rate among children aged 3–19 years was 37.7%. The study also found a close association between myopia prevalence and gender, and the analysis showed an increasing trend in myopia prevalence as the study years progressed (18). According to the data from the Chinese Ministry of Education for the 2019–2020 academic year, the prevalence of myopia among primary school students was 36.49%. Looking at other provinces and cities in China, the prevalence of myopia among primary school students is 37.7% in Hong Kong, and 33.6% in Chongqing (19, 20). In northern India, the prevalence of myopia among primary school children was reported to be 21.1%, in South Korea, the prevalence of myopia among children and adolescents aged 5–18 years reached as high as 65.4%. Another study conducted in Singapore revealed a myopia prevalence rate of 31.6% among primary school children. Interestingly, in Japan, a similar survey reported a significantly higher prevalence of myopia, with Japanese elementary school students reaching 76.5% (21–24). The prevalence of myopia is generally high in East Asia. Therefore, it is of the utmost importance to implement effective strategies for the early detection of myopia and the implementation of myopia prevention and control measures.

The findings of this study demonstrate a clear linear relationship between grade level and the prevalence of myopia, particularly in primary school grades where the prevalence of myopia escalates with higher grade levels. Numerous studies have consistently established a strong association between myopia prevalence and grade levels (25–28), which can be attributed to heightened academic pressures, prolonged close-eye use, diminished engagement in outdoor activities, and sleep decrease as students progress to higher grades. The changes in ocular parameters that contribute to refractive error predominantly occur during childhood (29–31). In addition, the impact of COVID-19 on education and lifestyle has led to an increase in near-work activities and screen time, accompanied by a decrease in outdoor activities. This has made regular eye examinations even more important in preparing for myopia management (32). The current research findings indicate a significant increase in the prevalence of myopia during the second semester of the academic year, with a particular emphasis on students in grades 1–3 and 7. Consequently, it is imperative to prioritize targeted interventions aimed at controlling and preventing myopia in these specific cohorts, namely lower-grade students and those who have recently transitioned to a higher-grade level. Such focused interventions hold significant potential for effectively curbing the escalating prevalence of myopia and its associated risks among school-aged children.

In Guangzhou, the annual incidence of myopia was between 20 and 30% in 2018 (33). Furthermore, in Anyang City, the yearly incidence of myopia increased from 7.8% in grades 1 and 2 to 25.3% in grades 5 and 6 (7). In our study, the overall incidence rate of myopia among primary and secondary school students within 6 months was 13.4%. Among primary school students, it was 11.9%, and among secondary school students, it was 15.4%. However, further analysis revealed that the incidence of myopia showed a linear increase with grade level in primary schools, while it tended to stabilize in different grades of secondary schools. This observation may be related to the education system in China and the developmental characteristics of children and adolescents themselves. The academic workload and visual stress for primary school students tend to increase as they progress to higher grades, accompanied by rapid changes in axial length. On the other hand, secondary school students generally have a heavier academic workload, but the changes in visual stress may be relatively smaller compared to primary school, and eye growth and development are more stable than in childhood. However, overall, the incidence of myopia in secondary school is still higher than that in primary school, which is consistent with the findings of the Mojiang Myopia Progression Study. This study reported that the annual incidence of myopia among first-grade primary school students was 33.6%, while it increased to 54% by the first grade of secondary school (5). These findings highlight the continued importance of implementing early myopia prevention strategies, with specific attention to the primary school stage, emphasizing the crucial need for sustained awareness and intervention measures during this critical period to tackle the risk factors linked to myopia development.

In our study, we analyzed the changes in refractive diopters over one semester in eyes with myopia and eyes without myopia. We have found that eyes with myopia exhibit a greater progression of myopia compared to eyes without myopia. This finding is consistent with the earlier viewpoint proposed by Mäntyjärvi (34), and can be expressed as follows: There exists a well-controlled controlled eye growth, but once the eyes cross the zero point and become myopic, the change in refractive error increases throughout the same period. As early as the late 1990s, highly competitive Hong Kong also observed this phenomenon. They reported that in a small sample of myopic eyes, the myopic progression over two and a half years in 6-year-old children was −1.92D, while non-myopic eyes showed only a −0.41D shift in refractive error during the same period (35). Our research findings indicate that among primary school students from Grade 2 to junior high school students in Grade 9, myopic eyes showed a myopic progression of −0.25D to −0.50D over one semester, while non-myopic eyes had no myopic shift (0D) during the same period. The results show a similar trend to the previously mentioned study conducted in Hong Kong. This further confirms the above viewpoint that once the eye alters into myopia, crossing over zero-point, its growth is no longer perfectly controlled and the axial length increase is getting out of control. Furthermore, we also investigated the changes in the degree of myopia among children who were already myopic. We found that, overall, there was a decrease in the prevalence of low myopia and an increase in the prevalence of moderate to high myopia in the second semester compared to the first semester among primary and secondary school students. When examining the data by grade level, this trend was observed from the fourth grade to the ninth grade. Another study that investigated the trends of myopia among primary and junior school students in the post-COVID-19 epidemic period over 3 years also reported the changes in the prevalence of different myopia degrees (36). From the data, it can be observed that the trend of the increasing prevalence of moderate to high myopia started in the fourth grade, which is consistent with the findings of our study. Nonetheless, our study, which investigated the changes over one semester, yielded the same outcomes. These findings emphasize the importance of timely implementing measures to control myopia, regulate its progression, and prevent a rapid worsening in its severity upon manifestation. Under the same educational background, it is important to adopt different myopia prevention measures for students in different grades of primary school, taking into account the school, family, and individual aspects. Moreover, Shanghai myopia research has found that well-implemented school myopia management can effectively reduce the risk of myopia among students. These measures mainly include encouraging outdoor activities during recess or physical education class providing adequate instruction in reading and writing postures, and good writing on blackboards, desks, and chairs with suitable heights (37).

### Limitations

This study differs from the majority of cross-sectional myopia screening studies as it adopts a prospective longitudinal design. Additionally, compared to the commonly used one-year observation period, our study has a more intensive and frequent observation period, and we have a sufficiently large sample size. However, there are certain limitations to this study. We employed non-cycloplegic refraction for myopia screening, as the use of cycloplegic agents in schools requires parental consent and may not be operationally feasible in large samples or school environments. This may have an impact on the detection of myopia. Nevertheless, the large sample size compensates for this limitation.

## Conclusion

In summary, our research indicates a high incidence of myopia among primary and secondary school students in Xuzhou, China, within one academic year. There is a significant increase in myopia prevalence within a single semester, accompanied by notable progression and a rise in high myopia rates. These issues worsen with higher grade levels. To effectively prevent and control myopia in children and adolescents, it is crucial to conduct two refractive examinations per year for early detection and intervention. Personalized prevention and control measures tailored to different grade levels are essential for effective management.

## Data availability statement

The original contributions presented in the study are included in the article/supplementary material, further inquiries can be directed to the corresponding author.

## Ethics statement

The studies involving humans were approved by Ethics Committee of Xuzhou Medical University Xuzhou Municipal Hospital, China. The studies were conducted in accordance with the local legislation and institutional requirements. Written informed consent for participation was not required from the participants or the participants’ legal guardians/next of kin because This is an observational study, and before conducting visual screening each semester, we inform parents and teachers about the screening’s purpose, methods, and the necessary precautions to take before the exam. We have obtained verbal consent from the parents, which was approved by the Ethics Committee of Xuzhou Municipal Hospital.

## Author contributions

LL: Conceptualization, Formal analysis, Investigation, Methodology, Project administration, Visualization, Writing – original draft, Writing – review & editing. YL: Conceptualization, Formal analysis, Methodology, Supervision, Writing – original draft, Writing – review & editing. QW: Methodology, Supervision, Writing – original draft, Writing – review & editing. MW: Methodology, Supervision, Writing – original draft, Writing – review & editing. WZ: Conceptualization, Methodology, Software, Supervision, Visualization, Writing – original draft, Writing – review & editing. XW: Conceptualization, Methodology, Software, Supervision, Writing – original draft, Writing – review & editing.
